# Reduced activation in empathy core regions during observation of social interactions in patients with borderline personality disorder: an fMRI-study

**DOI:** 10.1038/s41398-026-03989-5

**Published:** 2026-04-03

**Authors:** Vera Flasbeck, Björn Enzi, Georg Juckel, Martin Brüne

**Affiliations:** LWL University Hospital Bochum, Department of Psychiatry, Psychotherapy and Preventive Medicine, Ruhr University Bochum, Alexandrinenstraße 1-3, 44791 Bochum, Germany

**Keywords:** Human behaviour, Neuroscience

## Abstract

Patients with Borderline personality disorder (BPD) are known to exhibit aberrant empathy and heightened sensitivity to social threat. However, the neural mechanisms underlying these observations are not fully understood. In the present study, we therefore sought to assess empathy for somatic and psychological pain during a Social Interaction Empathy Task (SIET) in female patients with BPD (*n* = 50) and healthy participants (*n* = 55) during functional magnetic resonance imaging (fMRI). We further examined pain thresholds during a pain-pressure test (PPT) to the thenar muscles, self-harm behaviour, trait empathy, alexithymia and severity of BPD symptoms. Patients with BPD showed higher pain ratings for psychological pain and neutral conditions during the SIET, and higher pain ratings for psychological pain when rating from a first-person perspective, compared to a third-person perspective. The fMRI data revealed reduced activations in clusters including the right insula and hippocampus, bilateral superior and middle frontal gyri, left middle temporal gyrus, left pre-and postcentral gyri, left putamen and right anterior cingulum in patients with BPD. Activations of the left middle temporal gyrus when observing neutral scenarios correlated negatively with alexithymia and self-harm behaviour in the whole sample. In controls, middle temporal gyrus activation during viewing psychological pain was related to perspective taking (IRI), a capacity that was notably reduced in the patient group. Patients further exhibited elevated pain thresholds during the PPT and reduced pain intensity ratings for the right thenar. The findings indicated that patients with BPD showed altered processing of social interactions, speculatively due to deficits in perspective taking.

## Introduction

Borderline Personality Disorder (BPD) is a severe mental disorder that affects 1–6% of the general population in Western societies [1, 2]. It is characterized by instability in mood, relationships and self-image, as well as difficulties in emotion regulation, fear of being abandoned and chronic feelings of emptiness. These symptoms are often accompanied by increased impulsivity and self-harm behaviours [3, 4]. The development of BPD has been linked to insecure attachment patterns and maltreatment during childhood [5, 6]. Symptoms associated with BPD have in common that they impact an individual’s ability to function in social settings.

Researchers have utilized various methods to examine alterations in social functioning including the administration of questionnaires, behavioural tasks, and neuroimaging studies during emotion processing, empathy, or mentalisation tasks. Emotion recognition tests have revealed heightened sensitivity to negative facial expressions in BPD [7] and a tendency to perceive neutral or ambiguous emotions as negative [8, 9]. Furthermore, increased activation of the amygdala [10, 11] and reduced activation of the anterior cingulate cortex (ACC) [11] have been observed during facial emotion processing tasks in BPD. Patients were further shown to demonstrate heightened sensitivity to social pain, such as rejection during a ball-tossing game and showed higher activation of the ACC, medial prefrontal cortex (mPFC) and right precuneus during the task [12, 13]. Collectively, these findings indicate a heightened vigilance for emotional cues in BPD. Concerning the capacity to adopt the perspective and feel with others, studies on mentalising and empathy have yielded mixed results. Research has demonstrated that alterations in mentalising abilities, also referred to as Theory of Mind (ToM), show task and complexity dependent variations. Aberrant activations of brain regions implicated in ToM and empathy, such as the insula and amygdala, have been observed in BPD [14, 15].

Empathy research has primarily utilized empathy for pain tasks, which have been shown to activate the affective components of the pain matrix including the anterior cingulate cortex and the anterior insula. These region have also been identified as being activated during first-hand experiences of pain as well [16, 17]. In addition, the perspective participants have to adopt, i.e. self or first-person perspective and other or third-person perspective, has been demonstrated to influence pain ratings and brain activation levels [18]. In patients with BPD, previous trait empathy research reported increased scores of the personal distress scale on the Interpersonal Reactivity Index, a self-report questionnaire [19], which is considered a measure of emotional empathy. Furthermore, reduced scores on the cognitive perspective taking score have been frequently documented [20]. Other research suggests that patients with BPD exhibit heightened emotional contagion [21]. Dinsdale and Crespi interpreted the findings in BPD as a combination of impaired cognitive empathy and enhanced attention to, and perception of, social cues, together with dysfunctional higher-order processing involved in emotion regulation [22]. Utilizing an empathy for pain paradigm, which presented facial emotional expressions before presenting a human’s hand receiving painful stimulation, we showed differential activation of the right supramarginal gyrus and left anterior insula depending on the facial expression and pain condition (painful or control picture) only in the BPD group [23]. This finding aligns with the prevailing notion of hypersensitivity to social contexts and stimuli. In another study, we investigated empathy for somatic pain and empathy for psychological pain during the social interaction empathy task (SIET). We found increased pain ratings for psychologically painful social interactions as well as for neutral interactions. By examining pain ratings during trials with a first-person perspective and a third-person perspective trials, we further showed that patients rated the pain intensity higher for first-person perspective trials during psychologically painful conditions and lower for somatic pain when compared to the third-person perspective [24]. This could indicate that patients have heightened sensitivity to social and psychological pain, while experiencing reduced sensitivity to somatic pain when evaluating interactions from their own perspective. The altered rating of somatic pain could be probably due to frequently occurring self-injurious behaviour and increased pain thresholds reported in patients with BPD [25, 26]. Recently, these findings have been replicated in an Italian sample using an adapted version of the SIET [27]. Both studies suggest that alexithymia is associated with increased pain attribution to psychological pain in patients with BPD [24, 27]. However, whether differences in behaviour would be related to altered brain activation pattern has not been investigated.

In the present study, we therefore aimed to analyse the association of pain sensitivity, self-harm behaviour, severity of BPD symptoms, trait empathy, alexithymia and empathy for psychological and somatic pain (assessed by using the SIET) during fMRI in an independent sample. We sought to determine the activity in brain regions involved in the previously proposed hypersensitivity to social threat, especially for the psychological pain condition, and reduced sensitivity to somatic pain. Regarding psychometric characteristics, we expected to replicate previous data showing increased emotional empathy and reduced cognitive empathy, as well as higher scores for self-injurious behaviour, alexithymia, and BPD psychopathology in the patient group. Concerning behavioural investigations, we further expected to replicate previous research reporting higher pain thresholds for the BPD group and increased pain ratings for psychologically painful and neutral social interactions during the SIET. For the fMRI data, independent of group, we anticipated that psychological pain would activate the affective parts (ACC and anterior insula) of the pain matrix, regions implicated in mentalisation and the amygdala to a greater extent than somatic pain would. Neutral social interactions were proposed to show the lowest activation. We hypothesized altered brain activation in empathy-for pain core regions [17] in patients with BPD. Specifically, we predicted higher recruitment of the anterior cingulate cortex, the anterior insula, and the amygdala during the processing of psychological pain and neutral interactions, as previous studies have documented altered behaviour related to these task conditions. Additionally, we anticipated that the perspective might modulate brain activation pattern for neutral and psychological pain conditions in patients, given its impact on behavioural outcomes. Finally, we expected correlations of brain activity with trait empathy, alexithymia and borderline symptoms including self-injurious behaviour. Further exploratory correlation analyses were performed to study associations between behavioural data (pain-pressure test and SIET) and brain activations during the SIET.

## Methods and materials

### Study design and procedures

After screening for exclusion criteria, an appointment was scheduled with participants according to their menstrual cycle phase or hormonal intake. Participants could decide whether to complete several questionnaires prior to the appointment. On the study day, a diagnostic interview was conducted with healthy controls first, followed by the completion of questionnaires. After receiving task and MRI instructions, participants underwent fMRI scanning while completing the empathy task. Finally, the pain pressure test was performed. In addition to the measures described below, oxytocin levels and rejection sensitivity were assessed (data not shown here). Following the 21-word disclosure proposed by Simmons et al [28], we report how we determined our sample size, all data exclusions, all manipulations, and all measures reported in the present manuscript.

### Participants

Fifty patients with BPD were recruited during inpatient treatment at the LWL-University Hospital Bochum and diagnosed per DSM-5 criteria [3] as part of the routine clinical assessment during their inpatient treatment. Exclusion criteria included ADHD, bipolar disorder, psychotic disorders and current substance abuse. Comorbidities were depressive episodes (*n* = 14; 28%), post-traumatic stress disorder (*n* = 8; 16%), eating disorder (*n* = 4; 8%), substance abuse (*n* = 2; 4%) and an anxiety/phobic disorder (*n* = 1; 2%). Twelve patients were medication-free (24%), the rest received antidepressants (*n* = 23; 46%), antipsychotics (*n* = 1; 2%), antidepressants and antipsychotics combined (*n* = 13; 26%), anticonvulsants (*n* = 1; 2%) and additional anticonvulsants (*n* = 3; 6%). Fifty-five healthy controls (HC) were recruited online, with no mental disorders and psychotropic medication. Psychological health was verified via short diagnostic interviews (Mini-DIPS; [29]) based on DSM-5 criteria, by a trained interviewer. General exclusion criteria were neurological or severe somatic conditions and pregnancy. For MRI-safety, participants had to free of MRI-unsafe metals; tattoos had to lack iron-oxide ink. All participants were tested either during the hormonal contraception intake phase or the follicular phase (menstrual cycle day: patients 9.3 ± 4.3; HC 10.3 ± 4.5, *t*(60) = 0.85*, p* = 0.397) and needed sufficient German knowledge. Written informed consent was obtained. They received either 30 € or 2.5 test subject hours (for students of psychology, *n* = 16 in HC group). The authors assert that all procedures contributing to this work comply with the ethical standards of the relevant national and institutional committees on human experimentation and with the Helsinki Declaration of 1975, as revised in 2013. All procedures involving human subjects/patients were approved by the Ethics Committee of the Medical Faculty of the Ruhr-University Bochum (project number 20-6837). The sample size was chosen to be comparable to that of our previous SIET study (*n* = 50;48) and exceeded the commonly recommended minimum of approximately 30 participants for fMRI studies [30].

### Questionnaires

Handedness was assessed with the Edinburgh Handedness Inventory [31] (10 + 2 items). Verbal intelligence was measured using the Multiple Choice Vocabulary Intelligence Test (Mehrfachwahl-Wortschatz-Intelligenztest Version B; MWT-B; [32]). Pain sensitivity was evaluated via the Pain Sensitivity Questionnaire (PSQ; [33]) yielding “minor pain”, “moderate pain” scales and a total score. We investigated trait empathy using the Interpersonal-Reactivity-Index (IRI), which contains two cognitive subscales, perspective taking (PT) and fantasy (FS), and two affective subscales, empathic concern (EC) and personal distress (PD) [19, 34]. Alexithymia was measured with the Toronto Alexithymia Scale-20 (TAS-20 [35]; German Version [36]) and childhood maltreatment with the Childhood Trauma Questionnaire (CTQ; German Version [37]). Borderline symptoms were assessed using the Borderline Symptom List 23 (BSL-23; [38]). All participants further completed the Deliberate Self-Harm Inventory (DSHI; [39]; German version [40]), depressive symptoms were recorded by the Beck Depression Inventory II [41, 42]. For details, see supplemental information.

### Pain-pressure test (PPT)

Pain-pressure threshold, was measured using a pressure algometer with a 1 cm² round rubber tip (Force Dial FDK20; Wagner Instruments, Greenwich, USA) [43]. Pressure was applied to the thenar at 10 N/s, and participants indicated when pain began; testing then stopped. After a practice trial, three measurements were taken at varying thenar sites and averaged (in kPa). The PPT adhered to the standardized quantitative sensory testing (QST) protocol established by the German Research Network on Neuropathic Pain (DFNS) [44]. For pain evaluation, 10% was added to this average, and the resulting pressure was applied for 10 seconds. Participants rated pain intensity (0 = no pain to 10 = worst imaginable) and unpleasantness (0 = not unpleasant to 10 = most unpleasant imaginable). Threshold and pain evaluation were conducted on both hands in random order. One patient did not participate in the PPT, and in another patient the PPT was measured only on the left hand due to an injury to the right hand. In addition, one healthy control did not undergo the PPT, and for another healthy control participant, the right hand was not assessed due to extensive sports-related use of that hand. Consequently, the final analyses included *n* = 49/48 (left/right) patients with BPD and *n* = 54/53 (left/right) healthy controls.

### Social interaction empathy task

The paradigm was an fMRI-adapted version of a previously used empathy task (EEG and behavioural studies [24, 45–47]), presenting images of somatic and psychological pain in male–female social interactions, plus neutral scenes as controls (Figure S1; for details, see [24]). It includes 15 images (5 per condition: neutral, somatically painful, psychologically painful). Stimuli were shown via a monitor (mirror on head coil) or MRI-compatible LCD googles using Presentation® software (Neurobehavioral Systems, Inc., Version 17.2). Participants rated intensity of the pain using a scale (1 = not painful at all, to 5 = very painful) using an MRI-compatible keyboard, adjusting ratings during a response window. Trials alternated between third-person (estimating the woman’s pain) and first-person (estimating own pain in the situation) perspectives, cued by a slide shown before each trial. Because the “victim” in all scenarios was female, all participants were also female. After each block, participants rated how easily they could adopt the instructed perspectives and empathize. The experiment included 176 trials (30 per pain condition, 28 neutral per perspective) across two blocks, lasting ~40 minutes. Behavioural data (pain ratings 1–5) were analysed by stimulus condition. The final analysis included data from a total of 102 participants (n HC = 53, n BPD = 49) because one patient and two control participants did not conduct the SIET. In the BPD group, four patients did not complete both runs (155; 172; 135; 82 trials of 176 trials finished), and in the HC group, two did not complete both runs (88; 88 trials finished).

### fMRI scanning and data analysis

fMRI data were acquired on a 3 T Philips Achieva System with a 32-channel SENSE head coil. Scanning begun with a high-resolution T1-weighted anatomical scan (3D TFE: matrix 240 × 188 mm², reconstructed to 256 × 256 mm², FOV 240 × 188.8 × 220 mm^3^, in-plane resolution 1.0 × 1.0 mm², reconstructed to a final voxel size of 0.94 × 0.94 × 1.0 mm³, TR = 8.2 ms, TE = 3.7 ms, flip angle α = 8°, SENSE factor RRL = 2.5 and RFH = 2.0, 220 transverse slices). Functional data during the SIET were recorded using T2*-weighted echo-planar (EPI) interleaved slices, parallel to the bi-commissural plane. The BOLD (blood-oxygen level dependent) signal was obtained using a sensitivity encoded single-shot echo-planar imaging protocol (SENSE-sshEPI: number of slices 32, matrix 72 × 74 mm^2^, reconstructed to 112 × 112 mm², FOV 220 × 220 mm², in-plane resolution 3.0 × 3.0 mm², slice thickness 3 mm with 1 mm gap, reconstructed to a final voxel size of 1.96 × 1.96 × 3 mm^3^, TR = 2000 ms, TE = 35 ms, flip angle α = 86°, SENSE factor RAP = 2.0). The first five dummy scans were discarded. Two SIET runs (520 volumes each) lasted ~20 minutes. The total sample containing SIET data comprised 53 HC and 49 patients with BPD. One control participant did not accomplish the scanning procedure, and another four patients and two control participants did not complete both SIET scanning runs. Three of these incomplete datasets of patients and one of controls were excluded during preprocessing. Data of one control participant had to be excluded due to technical issues. Thus, *n* = 45 for the BPD group and *n* = 50 or the HC group were included into the final data analysis.

The fMRI data were preprocessed and analysed using SPM12 (Wellcome Trust Center for Neuroimaging, Institute of Neurology, University College London, UK; http://www.fil.ion.ucl.ac.uk) and MATLAB 2022b (The MathWorks Inc, Natick, MA, USA). Steps included slice timing correction, realignment, co-registration, normalization (SPM T1 template), and smoothing with an 8-mm FWHM Gaussian kernel (resampled voxel size: 2 × 2 × 2 mm³). A high-pass filter (100 s) was applied. Successful co-registration was manually checked for each dataset. Due to extensive movement, four patients and one healthy participant were excluded. Analyses focused on social situation onsets across pain conditions and perspectives. Realignment parameters were included as nuisance regressors. Single-subject models convolved condition onsets with a canonical hemodynamic response function [48]. Further statistical analysis followed the general model approach [49] for event-related fMRI studies. We calculated a mixed model ANOVA with the factors group (BPD/HC), perspective (first-person perspective/ third-person perspective) and condition (neutral/somatically painful/ psychologically pain). The initial voxel-level threshold was *p*_FWE_ < 0.05, extent k > 10 voxel. Activations were labelled via the AAL atlas [50] implemented in WFU PickAtlas [51]. Only regions surviving cluster-level FWE correction *p*_FWE_ < 0.001 were reported here and percent signal changes were extracted using MarsBar (http://marsbar.sourceforge.net/) [52] for further analysis in SPSS. For a list of all activations with a whole brain threshold of *p*_FWE_ < 0.001 see supplemental information Table S4. In addition to the aforementioned ANOVA, we performed exploratory comparisons between groups for the three experimental conditions in the “core empathy regions,” including the insula and the anterior cingulum.

### Statistical analysis

Demographical and psychometric group differences were assessed by *t*-tests, Mann-Whitney-U-tests and chi-squared tests. As pain thresholds and pain evaluation during the PPT were non-normally distributed, Mann-Whitney-U tests were selected for comparisons between groups. Behavioural data from the SIET were analysed via mixed-model ANOVA with factors group (BPD/HC), condition (neutral/ somatic pain/ psychological pain) and perspective (1^st^ person-perspective/ 3^rd^ person-perspective). Greenhouse Geisser corrected results are reported. Post-hoc comparisons used independent and dependent *t*-tests. Partial correlation coefficients were computed between psychometric questionnaires (BSL-23, TAS, DSHI and IRI) and brain activations during the SIET, controlling for education levels. We selected to focus on correlations with activations in the left middle temporal gyrus, as this region has been shown to be involved in group, condition and perspective effects. We applied Bonferroni correction to adjust for multiple comparisons. The aforementioned analyses were hypothesis-driven, whereas additional correlation analyses shown in the supplemental material were considered exploratory. The additional correlations were computed between brain activations, behavioural data, PPT variables and childhood trauma. All statistical analyses (*t*-tests, Mann-Whitney U tests, chi-squared tests, and correlations) were conducted using a two-tailed approach with an initial significance level of α = 0.05. We reported partial *ƞ²* and Cohen’s *d* as measures of effect size. SPSS 29 was used for statistical analysis (IBM Corp. Released 2023. IBM SPSS Statistics for Windows, Version 29.0.2.0 Armonk, NY: IBM Corp).

## Results

### Participants’ characteristics and psychometric scores

Patients with BPD did not differ significantly from controls with regard to age, IQ, handedness, language, marital status and use of hormonal contraception. However, the groups did differ in BMI, education, nicotine use and psychometric questionnaires (Table 1). Patients with BPD conducted more frequently and more different ways of self-harm behaviour (for details see Table S1). In the patients’ group, 42, i.e. 86% insured themselves within the last year.

**Table 1:** Demographic characteristics and psychometric questionnaire results of control participants and patients with BPD.

	HC group (*n* = 55)	BPD group (*n* = 50)	Statistics
Age (mean (*SD*))^c^	24.4 (4.3)	24.7 (4.9)	*t*(103) = –0.42, *p* = 0.66, *d* = –0.08
BMI (mean (*SD*))^c^	22.6 (2.3)	25.7 (7.7)	*t*(57.2) = –2.71, *p* = 0.009, *d* = –0.55
IQ (mean (*SD*))^a,c^	101.9 (12.1)	98.7 (11.1)	*t*(103) = 1.39, *p* = 0.166, *d* = 0.27
Handedness^b,c^ (mean (*SD)*; frequency left/right/both)	74.4 (43.2); 50/4/1	60.9 (47.6); 42/6/2	*t*(103) = 1.52, *p* = 0.132, *d* = 0.30
hormonal contraception (yes/no)	20/35	13/37	*χ²(*1) = 1.31, *p* = 0.253
Mother language (German/other/German+other)	38/10/5	45/3/2	*χ²(*2) = 5.56, *p* = 0.062
Marital status (single/divorced/relationship living together, relationship living apart/married	32/0/10/12/1	30/1/8/11/0	*χ²*(4) = 2.10, *p* = 0.718
Education (Certificate of Secondary Education/General Certificate of Secondary Education/ higher education entrance qualification/university degree/other)	0/1/37/17/0	5/16/25/2/2	*χ²*(4) = 34.24, *p* < 0.001
Nicotine use (no/low/high consumption)	52/3/0	21/9/20	*χ²(*2) = 36.01, *p* < 0.001
**Psychometric questionnaires** (mean (*SD*); median)^d^			
BSL-23 mean	0.2 (0.2); 28.1	2.2 (0.6); 80.4	*U* = 3.0, *Z* = –8.81, *p* < 0.001
BDI sum	4.6 (5.5); 28.4	37.2 (10.7); 80.1	*U* = 20.5, *Z* = –8.71, *p* < 0.001
CTQ emotional abuse	7.9 (3.7); 31.3	18.2 (5.5); 76.9	*U* = 182.0, *Z* = –7.68, *p* < 0.001
CTQ physical abuse	5.5 (1.9); 39.9	8.6 (4.7); 67.4()	*U* = 654.5, *Z* = –5.47, *p* < 0.001
CTQ sexual abuse	5.2 (0.9); 42.8	9.2 (6.1); 64.3	*U* = 811.0, *Z* =–4.71, *p* < 0.001
CTQ emotional neglect	8.6 (3.9); 32.3	16.9 (4.4); 75.8	*U* = 236.5, *Z* = –7.32, *p* < 0.001
CTQ physical neglect	6.4 (2.5); 37.3	9.9 (3.8); 70.3	*U* = 513.0, *Z* = –5.68, *p* < 0.001
PSQ minor	3.0 (1.6); 56.2	2.6 (1.5); 49.5	*U* = 1200.5, *Z* = –1.12, *p* = 0.263
PSQ moderate	5.1 (1.8); 57.5	4.6 (1.6); 48.0	*U* = 1126.0, *Z* = –1.60, *p* = 0.110
PSQ total	4.0 (1.6); 57.4	3.6 (1.4); 48.2	*U* = 1135.0, *Z* = –1.54, *p* = 0.124
TAS difficulties identifying feelings	12.2 (4.2); 29.6	24.8 (5.1); 77.2	*U* = 115.5, *Z* = –8.04, *p* < 0.001
TAS difficulties describing feelings	10.5 (4.3); 34.3	17.5 (4.4); 72.2	*U* = 366.5, *Z* = –6.41, *p* < 0.001
TAS externally oriented thinking	15.3 (3.8); 43.5	17.7 (4.3); 62.3	*U* = 862.5, *Z* = –3.18, *p* < 0.001
IRI perspective taking	20.1 (4.3); 60.4	16.4 (6.3); 43.7	*U* = 914.0, *Z* = –2.83, *p* = 0.005
IRI fantasy	17.8 (5.6); 46.7	19.7 (5.4); 59.0	*U* = 1028.5, *Z* = –2.08, *p* = 0.037
IRI empathic concern	21.7(6.8); 51.1	21.1 (5.2); 54.1	*U* = 1270.0, *Z* = –0.51, *p* = 0.613
IRI personal distress	12.2 (3.8); 29.1	22.1 (3.0); 78.8	*U* = 60.0, *Z* = –8.399, *p* < 0.001
Total number of DSHI-items answered with yes	0.46 (0.99), 27.95	7.04 (2.93),79.01	*U* = 24.5, *Z* = –8.91, *p* < 0.001

*BDI* beck depression inventory, *BSL*-23 borderline symptom list 23, *CTQ* childhood trauma questionnaire, *DSHI* deliberate-self-harm inventory, *IRI* interpersonal-reactivity-index, *PSQ* pain sensitivity questionnaire, *TAS* Toronto alexithymia scale.

^a^Multiple Choice Vocabulary Intelligence Test.

^b^Laterality quotient.

^c^ differences between groups calculated by t-tests.

^d^ differences between groups calculated by Mann-Whitney U tests.

### Pain pressure test

**Fig. 1:**
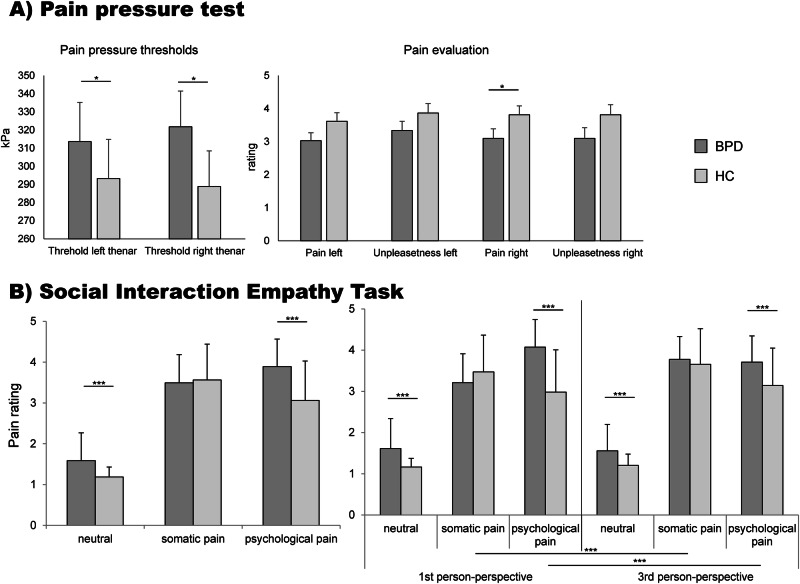
Behavioural results of the pain pressure test and the SIET. Averages of pain pressure thresholds (left) and pain as well as unpleasantness ratings (right) in panel **A** and pain ratings of the Social Interaction Empathy Task in panel **B**, both in patients with BPD (dark grey) and healthy controls (light grey). Error bars represent SEMs (**A**) and SDs (**B**). Significant differences between groups are indicated by * *p* < 0.05, *** *p* < 0.001.

Healthy participants reported that the sensations become painful at 293.2 kPa (mean; *SD* = 158.8) for the left thenar and at 288.9 kPa (mean; *SD* = 142.8) for the right thenar during the pain pressure threshold measurement. For patients with BPD, the thresholds were 313.6 kPa (mean; *SD* = 83.0) for the left thenar and 321.8 kPa (mean; *SD* = 104.2) for the right thenar. The thresholds differed significantly between groups, with higher thresholds in patients (threshold left thenar: *U* = 1016.0, *Z* = −2.03, *p* = 0.043; threshold right thenar: *U* = 962.5, *Z* = −2.11, *p* = 0.035). Patients further reported reduced pain for the right thenar, whereas unpleasantness ratings did not differ between groups (left thenar pain rating: HC *M* = 3.6 (1.9), BPD *M* = 3.0 (1.7), *U* = 1103.0, *Z* = −1.46, *p* = 0.146; left thenar unpleasantness rating: HC *M* = 3.9 (2.1), BPD *M* = 3.3 (1.9), *U* = 1175.0, *Z* = −0.98, *p* = 0.328; right thenar pain rating: HC *M* = 3.8 (1.9), BPD *M* = 3.1 (2.0), *U* = 965.5, *Z* = −2.09, *p* = 0.037; right thenar unpleasantness rating: HC *M* = 3.8 (2.2), BPD *M* = 3.1 (2.2), *U* = 965.5, *Z* = −1.10, *p* = 0.273; Fig. 1A).

### Social interaction empathy task – behavioural findings

The mixed-model ANOVA revealed main effects for condition (*F*(1.98,198.04) = 435.91, *p* < 0.001, partial *ƞ²* = 0.81), perspective (*F*(1.0,100.0) = 15.73, *p* < 0.001, partial *ƞ²* = 0.136) and group (*F*(1.0,100.0) = 15.70, *p* < 0.001, partial *ƞ²* = 0.136). Significant interactions occurred for condition x group (*F*(1.98) = 14.79, *p* < 0.001, partial *ƞ²* = 0.129), perspective x condition *F*(1.71,170.53) = 43.24, *p* < 0.001, partial *ƞ²* = 0.30) and perspective x condition x group (*F*(1.71) = 34.08, *p* < 0.001, partial *ƞ²* = 0.25). To start with effects regarding group differences, the significant main effect of group shows that patients rated the pictures in average as more painful compared to controls (pain ratings mean: BPD = 2.99 (0.45), HC = 2.60 (0.53), *t*(100) = −3.96, *p* < 0.001, *d* = −0.79; Fig. 1B).

The interaction condition x group indicated that patients with BPD rated neutral and psychologically painful situation more painful than control participants, whereas no group differences occurred for somatically painful conditions (neutral: BPD = 1.59 (0.66), HC = 1.18 (0.23), *t*(58.93) = −4.02, *p* < 0.001, *d* = −0.82; psychological pain: BPD = 3.89 (0.60), HC = 3.06 (0.96), *t*(88.49) = −5.29, *p* < 0.001, *d* = −1.03; somatic pain: BPD = 3.49 (0.56), HC = 3.56 (0.86), *t*(89.70) = 0.49, *p* = 0.627, *d* = 0.10). The perspective x condition x group interaction showed that patients with BPD evaluated psychological pain higher when considering the pain for themselves, compared to 3^rd^ person-perspective. In contrast, healthy controls rated psychological pain higher in the 3^rd^ compared to the 1^st^ person perspective (see Table S2). Between-group comparisons further revealed that ratings for neutral and psychologically painful conditions differed between groups in both perspectives, whereas no differences were found for somatic painful conditions in either perspective. For all those conditions, patients reported higher pain ratings. The main effect of perspective and the interaction of condition x perspective are shown in the supplemental information (Table S3).

### fMRI data

**Fig. 2:**
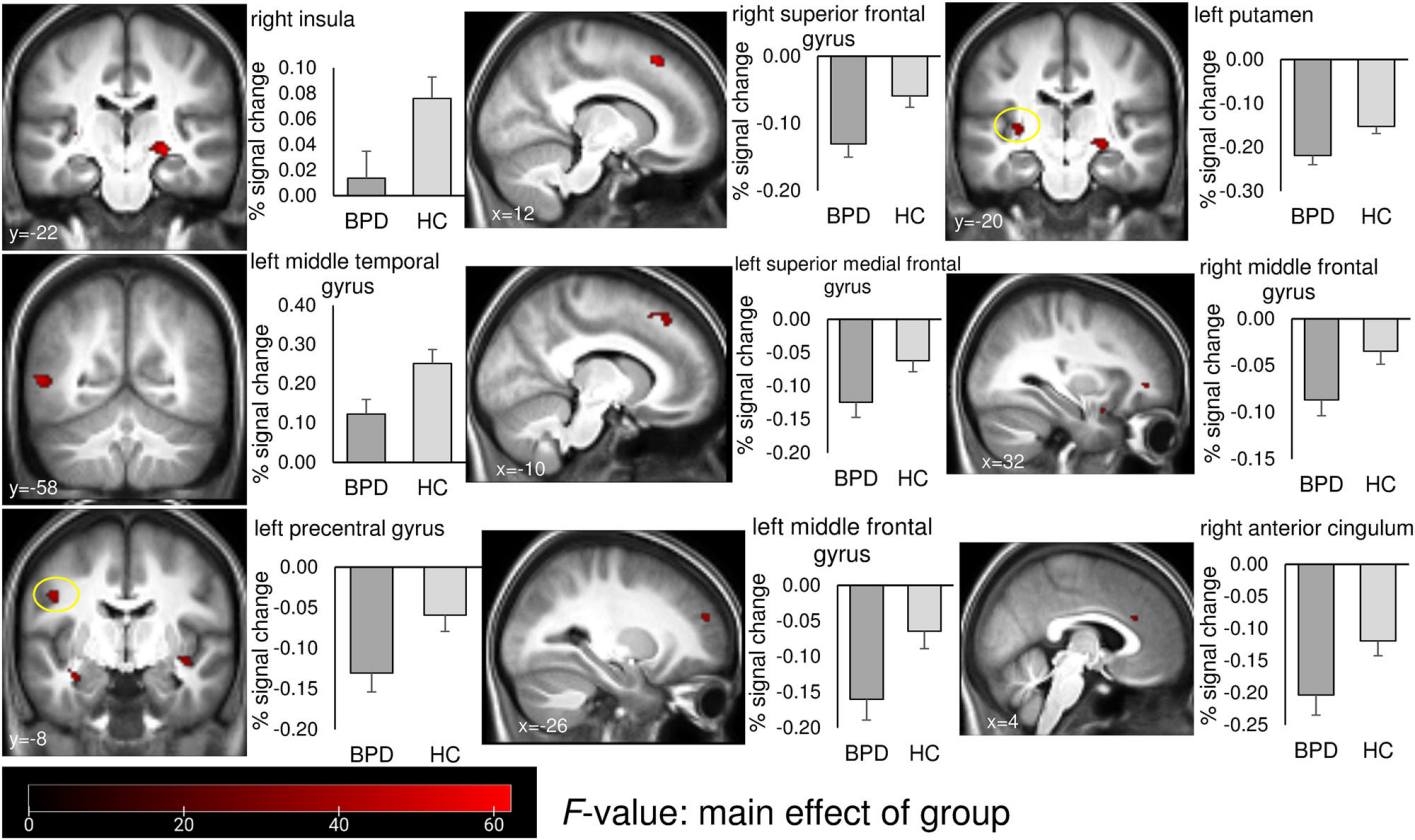
Main effect of group on brain activation during the empathy task. Activations in brain regions showing differences between patients with BPD and healthy controls with *p*_FWE_ < 0.001 and k > 8 voxel for visualization. Error bars = SEM.

The 2 × 2 × 3 ANOVA with factors group (BPD/HC), perspective (1^st^ person-perspective/ 3^rd^ person perspective) and condition (neutral/ somatic pain/ psychological pain) revealed a main effect of group, but no further interaction with group. The activations for the main effect of group are presented in Table 2 and Fig. 2.

**Table 2:** Brain activations during the SIET for the main effects of group and condition.

Main effect	Region	Hemisphere	Coordinates (MNI)	Extent (voxel) region (total cluster)	*F* (peak)	equiv*Z* (peak)
Group						
	Insula/ Hippocampus	R	24 −22 −6	120/ 97(679)	62.15	7.58
	Superior frontal gyrus	R	12 24 52	84 (141)	58.60	7.37
	Middle temporal gyrus	L	−54 −58 6	115 (116)	49.24	6.77
	Precentral gyrus/ Postcentral gyrus	L	−46 −8 32	89/ 83(195)	43.85	6.39
	Superior medial frontal gyrus/ Superior frontal gyrus	L	−10 34 48	68/66 (154)	41.01	6.18
	Middle frontal gyrus	L	−26 50 22	46 (71)	40.78	6.16
	Putamen	L	−32 −20 4	27 (191)	39.61	6.07
	Middle frontal gyrus	R	32 44 −2	6 (61)	37.77	5.93
	Anterior cingulum	R	4 28 26	95 (137)	34.45	5.66
Condition						
	Supramarginal gyrus	L	−60 −28 28	307 (468)	52.30	65535.00
	Middle temporal gyrus	L	−50 −62 −4	74 (109)	25.79	6.61
	Cuneus	L	−18 −60 18	78 (154)	23.94	6.35
	Precuneus/Calcarine sulcus	R	20 −60 20	53/51 (133)	23.62	6.31
	inferior triangular part of the frontal gyrus	L	−48 24 4	73 (73)	21.22	5.95
	inferior parietal lobule	L	−40 −42 50	134 (180)	19.67	5.71
	Precuneus	L	−2 −54 26	105 (168)	18.00	5.44

Initial threshold *p*_FWE_ < 0.05 for k > 10 voxel. All reported activations survived FWE correction on cluster level with *p*_FWE_ < 0.001. Only regions contributing most to the clusters are listed, cluster sizes for those regions are provided as well as total cluster sizes with initial thresholds.

**Fig. 3:**
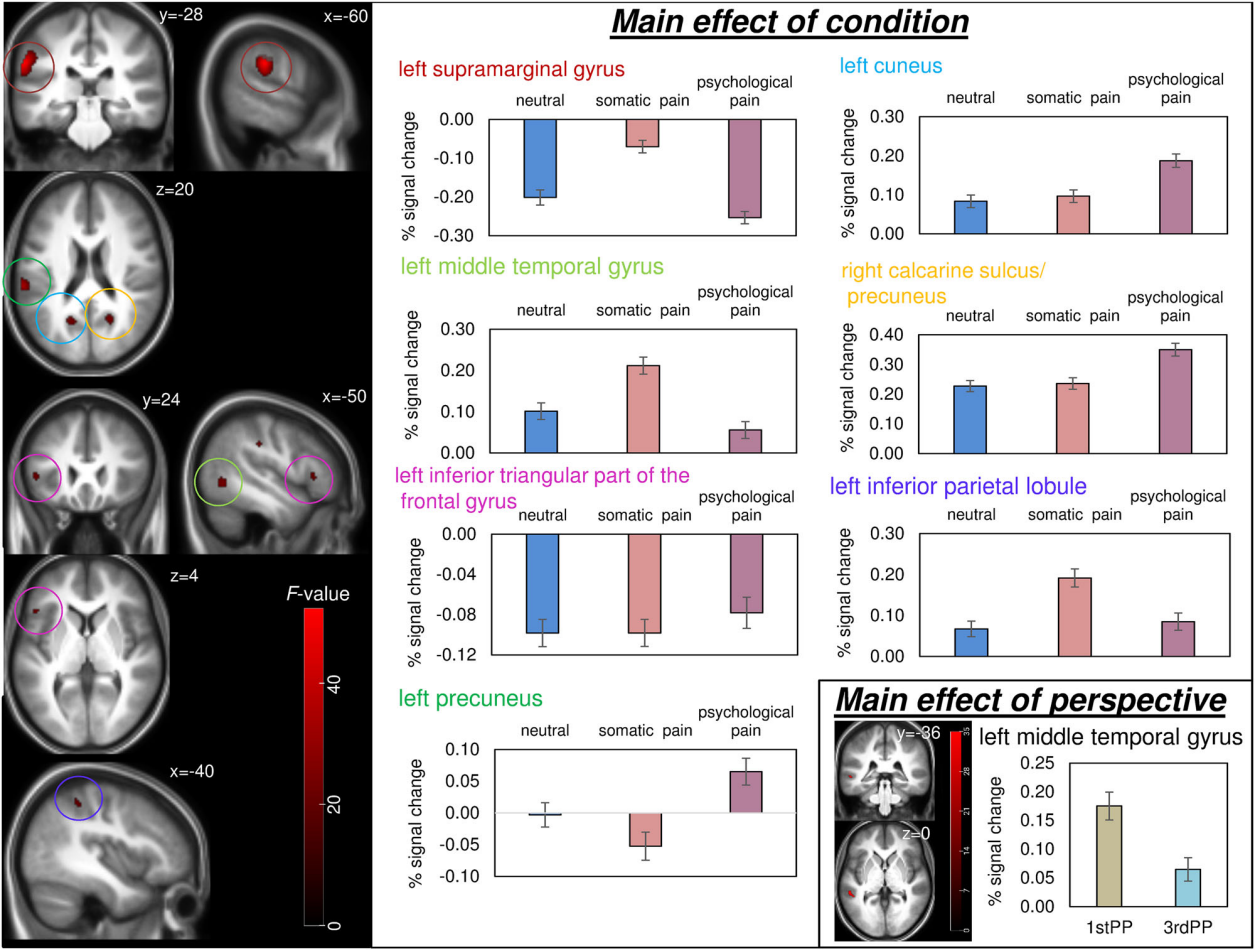
Main effects of condition and perspective on brain activation during the empathy task. Activations in brain regions showing the main effects of condition (perspectives and groups averaged) and perspective for *p*_FWE_ < 0.001 and k > 10 voxel for visualization. Different brain regions are indicated by different colours. Error bars = SEM.

Patients with BPD showed overall reduced brain activation during the empathy task. The main effect of condition showed differential brain activation in regions listed in Table 2 and Fig. 3. In the supramarginal gyrus, the cuneus, the precuneus and calcarine sulcus, the highest activation (deactivation for supramarginal gyrus) occurred during the presentation of psychologically painful social interactions. In the temporal (middle) and parietal (inferior) gyri, somatically painful images yielded the highest activation compared to the two other conditions.

The main effect of perspective revealed activation of the left middle temporal gyrus (-56 -36 0; *F* = 34.98, equiv*Z* = 5.71; extent 166 voxel, total cluster extent 232 voxel) indicating higher activation during the 1^st^ person-perspective trials when compared to 3^rd^ person-perspective trials (Fig. 3).

We processed exploratory comparisons between groups for pain conditions in the “core empathy regions” insula and anterior cingulum, as revealed by the main effect of group. As regards the right insula, patients with BPD showed lower activations for all conditions compared to the control group (neutral BPD = 0.01 (0.13), HC = 0.09 (0.11), *t*(93) = 3.57, *p* < 0.001, *d* = 0.73; somatic pain BPD = −0.03 (0.13), HC = 0.07 (0.13), *t*(93) = 3.83, *p* < 0.001, *d* = 0.79; psychological pain BPD neutral BPD = 0.01 (0.12), HC = 0.08 (0.12), *t*(93) = 3.19, *p* = 0.002, *d* = 0.66). For the right anterior cingulum, lower activation occurred for neutral and psychologically painful interaction in patients with BPD (neutral BPD = −0.24 (0.18), HC = −0.11 (0.16), *t*(93) = 3.64, *p* < 0.001, *d* = 0.75; psychological pain BPD = −0.18 (0.18), HC = −0.11 (0.14), *t*(93) = 2.04, *p* = 0.044, *d* = 0.42). No difference between groups occurred for somatically painful conditions (somatic pain BPD = −0.19 (0.20), HC = −0.13 (0.12), *t*(72.9) = 1.73, *p* = 0.088, *d* = 0.36).

### Correlations

**Fig. 4:**
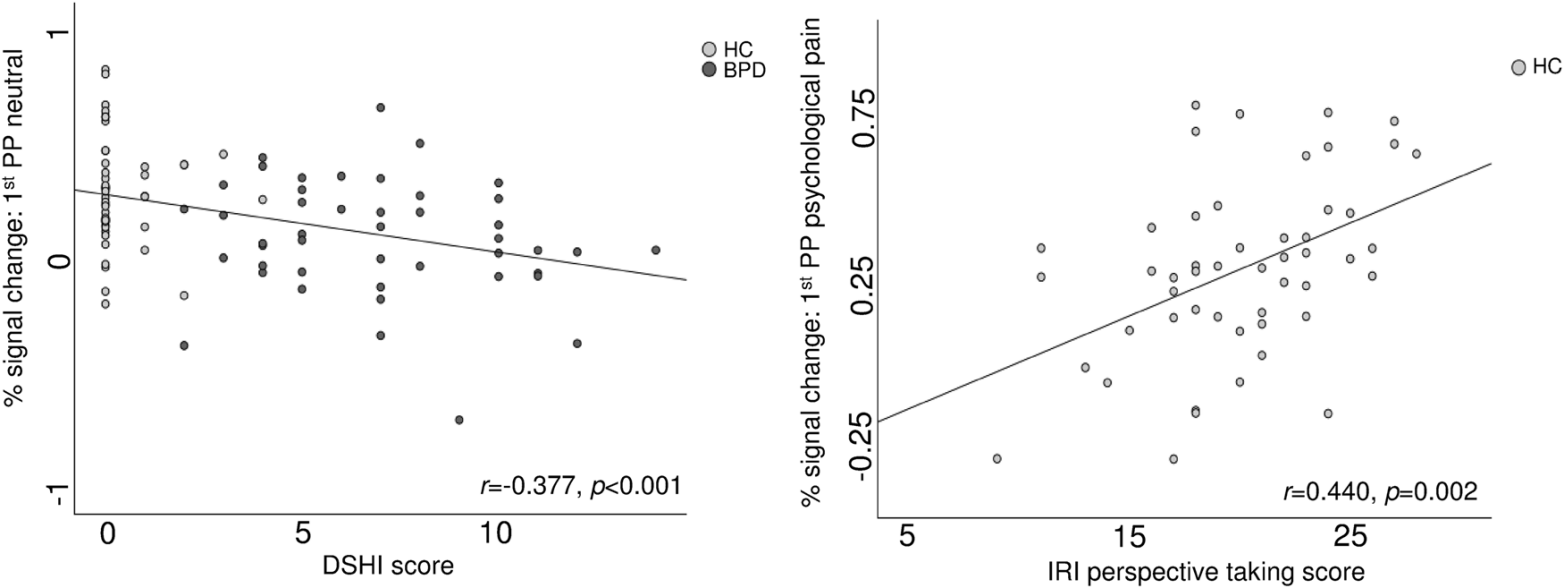
Correlations between SIET-related brain activation in the left middle temporal gyrus and psychometric measures. Left panel: Activation for the first-person perspective neutral condition correlated with self-injurious behaviour (DSHI). Right panel: Activation for the first-person perspective psychological pain condition correlated with perspective taking (IRI).

We calculated correlational analyses between activations during the SIET and psychometric questionnaires for left middle temporal gyrus, as this region showed to be involved in group, condition and perspective effects (Table 3). In the whole sample, correlations between activations during viewing neutral interactions and alexithymia (difficulties identifying feeling; for the 1^st^ person-perspective) and self-harm behaviour (1^st^ and 3^rd^ person-perspectives) survived correction for multiple testing (Bonferroni correction for 6 regions x 4 measures, i.e. 24 tests; *p* ≤ 0.0021). Within the control group, higher activation during viewing psychological painful interactions in the 1^st^ person-perspective was related to higher perspective taking capacity. Within patients, no correlations remained significant after Bonferroni correction. The most relevant correlations are represented in Fig. 4.

**Table 3:** Correlations (*r*(*p*)) between brain activations of left middle temporal gyrus during the SIET and psychometric questionnaires in the whole sample as well as HC and BPD groups separately, controlled for education levels. Significant correlations are marked by bold font, correlations surviving Bonferroni correction are marked with additional * (*p* ≤ 0.002).

Condition/questionnaire	TAS DIF	TAS DDF		TAS EOT	BSL-23	DSHI	IRI PT	IRI FS	IRI EC	IRI PD
**Whole sample**										
**1**^**st**^ **person perspective**										
neutral	−**0.364** ( < 0.001)*	−**0.271** (**0.011)**		−**0.223** (**0.037)**	−**0.305** (**0.004)**	−**0.377** ( < 0.001)*	**0.250** (**0.019)**	−0.208 (0.052)	0.049 (0.653)	−**0.312** (**0.003)**
somatic pain	−0.183 (0.089)	−**0.238** (**0.026)**		−0.188 (0.079)	−0.176 (0.101)	−0.188 (0.080)	0.125 (0.248)	−0.140 (0.192)	−0.034 (0.751)	−0.209 (0.051)
psychological pain	−**0.232** (**0.030)**	−0.192 (0.073)		−0.141 (0.189)	−0.159 (0.138)	−**0.210** (**0.050)**	**0.239** (**0.025)**	−0.140 (0.194)	0.043 (0.688)	−**0.223** (**0.037)**
**3**^**rd**^ **person perspective**										
neutral	−**0.248** (**0.020)**	−0.163 (0.128)		−0.180 (0.094)	−**0.231** (**0.030)**	−**0.325** (0.002)*	0.203 (0.058)	−0.152 (0.158)	0.019 (0.858)	−**0.235** (**0.028)**
somatic pain	−**0.255** (**0.016)**	−**0.254** (**0.017)**		−0.130 (0.229)	−**0.230** (**0.031)**	−**0.265** (**0.013)**	0.170 (0.114)	−0.096 (0.374)	−0.024 (0.823)	−**0.292** (**0.006)**
psychological pain	−**0.240** (**0.024)**	−0.182 (0.089)		−**0.236** (**0.027)**	−0.158 (0.142)	−**0.280** (**0.008)**	**0.223** (**0.037)**	−0.003 (0.979)	0.069 (0.525)	−0.204 (0.056)
**HC group**										
**1**^**st**^ **person perspective**										
neutral	−0.052 (0.736)	−0.251 (0.096)	−0.191 (0.208)		−0.221 (0.144)	−0.065 (0.673)	**0.339** (**0.023)**	−0.225 (0.138)	0.010 (0.947)	−0.191 (0.208)
somatic pain	−0.018 (0.905)	−0.132 (0.386)	−0.147 (0.336)		−0.277 (0.065)	−0.025 (0.871)	**0.368** (**0.013)**	−0.248 (0.101)	−0.068 (0.658)	−0.244 (0.106)
psychological pain	−0.043 (0.778)	−0.158 (0.301)	−0.170 (0.263)		−**0.404** (**0.006)**	−0.159 (0.296)	**0.440** (0.002)*	−0.168 (0.272)	−0.115 (0.454)	−0.258 (0.087)
**3**^**rd**^ **person perspective**										
neutral	−0.032 (0.833)	−0.147 (0.337)		−0.160 (0.292)	−0.250 (0.098)	−0.094 (0.537)	**0.298** (**0.047)**	−0.162 (0.287)	−0.020 (0.894)	−0.141 (0.356)
somatic pain	−0.010 (0.950)	−0.147 (0.336)		−0.095 (0.533)	−0.264 (0.080)	−0.127 (0.404)	0.271 (0.071)	−0.111 (0.469)	−0.062 (0.688)	−**0.303** (**0.043)**
psychological pain	−0.068 (0.657)	−0.162 (0.288)		−0.240 (0.112)	−**0.411** (**0.005)**	−0.124 (0.417)	**0.404** (**0.006)**	0.042 (0.786)	−0.005 (0.974)	−0.221 (0.145)
**BPD group**										
**1**^**st**^ **person perspective**										
neutral	−0.301 (0.053)	0.115 (0.470)		−0.061 (0.700)	0.036 (0.822)	−0.293 (0.060)	0.087 (0.586)	−0.197 (0.212)	0.083 (0.601)	0.046 (0.772)
somatic pain	−0.281 (0.071)	−0.297 (0.056)		−0.158 (0.319)	−0.122 (0.441)	−0.265 (0.089)	−0.133 (0.401)	−0.013 (0.937)	0.005 (0.974)	−0.065 (0.681)
psychological pain	−**0.343** (**0.026)**	−0.085 (0.593)		−0.020 (0.898)	0.063 (0.690)	−0.205 (0.192)	0.040 (0.802)	−0.106 (0.505)	0.189 (0.232)	−0.046 (0.774)
**3**^**rd**^ **person perspective**										
neutral	−0.195 (0.215)	0.105 (0.506)		−0.095 (0.552)	−0.006 (0.968)	−**0.340** (**0.027)**	0.044 (0.782)	−0.131 (0.410)	0.029 (0.854)	−0.027 (0.866)
somatic pain	−0.246 (0.117)	−0.112 (0.480)		0.007 (0.963)	0.004 (0.978)	−0.198 (0.210)	0.027 (0.866)	−0.078 (0.625)	0.019 (0.906)	−0.014 (0.932)
psychological pain	−0.257 (0.100)	−0.001 (0.994)		−0.141 (0.372)	0.155 (0.326)	−**0.331** (**0.032)**	0.036 (0.822)	−0.034 (0.829)	0.131 (0.407)	0.060 (0.704)

df whole sample = 86, df HC = 43, df BPD = 40.

*BSL*-23 borderline symptom list 23, *DSHI* deliberate self-harm inventory, *IRI* interpersonal reactivity Index (*PT* perspective taking, *FS* fantasy, *EC* empathic concern, *PD* personal distress), *TAS* Toronto alexithymia scale (*DIF* difficulties identifying feelings, *DDF* difficulties describing feelings, *EOT* externally oriented thinking).

We further calculated exploratory correlations between activations extracted from the main effect of condition in the whole sample and childhood maltreatment. Here, significant correlations emerged especially between activation of inferior triangular part of the frontal gyrus during viewing psychologically painful interactions and childhood maltreatment (see supplementary information Table S5).

Regarding associations of brain activations with SIET behaviour, in the whole sample, the most relevant correlations have been found for the left putamen, showing inverse correlations with pain ratings for psychological pain (Tables S6 and S7). When focusing on correlations between fMRI and PPT data, correlations reached relevant significance for pain ratings during the PPT and brain activations in the right superior frontal gyrus during painful conditions for the whole sample (Table S8). In general, the strength of the correlations was diminished in the subgroups, with more distinct associations evident in the control group.

## Discussion

In this study, we investigated brain activity correlates of empathy for pain using a validated paradigm previously applied in behavioural and EEG studies. Our aim was to characterize neural alterations underlying empathy deficits in BPD. Behaviourally, BPD patients rated psychological pain and neutral social interactions as more painful during the SIET than healthy controls, consistent with prior findings [24, 27]. We did not find significant differences between the groups in empathy for somatic pain, which could indicate a lack of statistical support or unaltered empathy for somatic pain. Besides, the findings could be interpreted to support the notion that patients with BPD might be selectively more sensitive towards negative social stimuli and tend to interpret neutral situations as negative [9, 14, 21, 22]. This hypersensitivity has been linked to enhanced resonance with others, stemming from an impaired self-other distinction, which culminates in elevated distress in patients [53]. This is further supported by increased personal distress scores on the IRI. In contrast, perspective taking was lower in the patients group, suggesting reduced cognitive empathy. These differences have been frequently reported in the literature [20] and in our own previous works [24, 54]. As the SIET yielded similar behavioural results in three different samples, one could carefully suggest that the task may be able to provoke altered empathy behaviour associated with BPD.

In addition to the behavioural studies conducted using the SIET, the present study further sought to examine the brain activation patterns underlying altered behavioural results observed in patients with BPD. fMRI analyses showed decreased activation in BPD patients across task conditions in clusters including the right insula/ hippocampus, bilateral superior and middle frontal (med) gyrus, left middle temporal gyrus, left precentral/ postcentral gyrus, left Putamen and right anterior cingulum. Superior and middle frontal cortex activation has been related to mentalising [55, 56], while insula and ACC were consistently engaged during empathy for pain tasks [17, 55, 57], which were less activated in patients in our study. Exploratory comparisons revealed lower activation of the right anterior cingulum for neutral and psychologically painful interactions in patients with BPD, but no difference for somatic pain. This finding underscores the significance of social threat for patients with BPD, suggesting that social interaction processing is not globally impaired. As the control condition, i.e. the neutral condition, also showed a social interaction, which has been rated as more painful by patients compared to controls, no group* condition interaction emerged since all conditions were processed differently in patients. Reduced activation may reflect impaired mentalising, perspective taking, or emotion regulation during observation of social interactions. Accordingly, altered activity of the ACC and insula has been reported during emotional scene processing and emotion regulation [58]. Previous findings regarding brain activations during empathy tasks in BPD are inconsistent due to high variations in tasks employed and are frequently related to emotion recognition.

The main effect of condition identified for clusters including the left supramarginal and middle temporal gyri, left cuneus, bilateral precuneus extending to the right calcarine sulcus and the left parietal lobule, has indicated differential brain activation depending on the social condition. More detailed, increased deactivation during neutral and psychological pain occurred in the left supramarginal gyrus. Psychological pain elicited highest activation in the left cuneus, the right calcarine and bilateral precuneus, whereas somatic pain was strongest in the left middle temporal gyrus and the left inferior parietal lobule. This paradigm is the first to directly compare psychological and somatic pain empathy within one task; previous research focused mostly on somatic pain or social exclusion. Our data shows that psychological pain and somatic pain in social interactions are processed differently. A further distinction was observed between both types of pain and neutral conditions, especially for the left supramarginal gyrus and the left middle temporal gyrus. Notably, the supramarginal gyrus is well-known for its role in somatic pain empathy [17, 57]. When pooling the data sets of BPD patients and controls, we found correlations between pain ratings and brain activations during task performance. The largest effect was observed in the left putamen, indicating that high activity correlated with low pain rating for psychologically painful interactions (see supplementary information Tables S6 and S7). This observation suggests a potential role for the putamen in modulating the response to potentially socially threatening stimuli, which may not be specific to either group (BPD vs. controls).

Regarding perspective, first-person trials showed greater left middle temporal gyrus activation than third-person trials. Previous work links perspective-taking to activity in regions including the supramarginal gyrus, superior temporal sulcus, secondary somatosensory cortex, ACC, insula, posterior cingulate/precuneus, and right temporo-parietal junction, mostly using simple pain stimuli [59]. Complex social interactions, as used here, may elicit differing activations. Given that perspective had a profound impact on behavioural outcomes, with patients with BPD evaluating psychological pain higher and somatic pain lower when considering the pain for themselves, modulation of fMRI data by perspective and condition was expected. However, as no interaction with perspective or condition emerged, it is possible that the study may have been underpowered to discover interactions at the neural level. To assess the role of perspective more thoroughly, we calculated correlations between pain ratings and brain activations, based on the main effects. This approach, however, also bears limitations. However, the findings were inconclusive and did not survive statistical correction for multiple testing (Table S7).

Pain sensitivity did not differ between groups on the PSQ questionnaire, but BPD patients had higher pain thresholds in the PPT, confirming earlier reports [25, 60, 61]. Reduced pain sensitivity has been related to self-injurious behaviour [26, 62], which also occurred in our sample. In neuroimaging studies, patients with BPD and self-injurious behaviour showed selective functional alterations in brain regions constituting emotional processing, such as the ACC and amygdala, executive functions, and reward processing during first-hand pain processing [63]. Beyond acute pain, patients with BPD may show increased chronic pain incidence and altered endogenous opioid system functioning [64]. The authors further concluded that altered cognitive and affective processing of pain, as well as emotional dysregulation might be causal for dysregulation of somatic pain, whereas sensory processing remains largely unaffected. Here, subjective pain ratings during PPT correlated most strongly with the activation of the right superior frontal gyrus during the observation of painful scenes (Supplemental material Table S8; *p* = 0.003). This finding suggests a potential association between affective pain evaluation and empathic responsiveness to others’ pain, i.e., the affective processing of pain related to the self and to others. However, as the correlations emerged in the whole sample, this cannot be considered a finding specifically relevant to BPD.

In the present study, we further aimed to investigate the factors that modulate empathy in patients with BPD by assessing correlations between brain activation and psychometric measures. To more thoroughly assess the roles of condition and perspective, we focused on correlations with activation in the left middle temporal gyrus, as this region has been shown to be involved in group, condition, and perspective effects. First, in the whole sample, inverse correlations were found between activations during the viewing of neutral interactions and the difficulties identifying feelings (TAS score indicating alexithymia) for the first-person perspective and self-harm behaviour (both perspectives). This could be interpreted as suggesting that alexithymia and self-harm behaviour may reduce brain activation in the left middle temporal gyrus, whose activity was higher in controls. The reduced activation could be associated with decreased processing of the social situation, which might further be related to higher social threat or pain attribution. This idea could be supported be correlations between brain activation and pain ratings for neutral conditions for this region (Supplemental material Tables S6 and S7). However, since the correlations would not survive correction for multiple testing, future research is required. In the BPD group alone, the correlations with psychometric scores did not survive correction for multiple testing; therefore, these associations would not be considered specific to BPD. Second, in healthy participants alone, higher perspective taking was related to higher activation during the viewing of psychologically painful interactions in the first-person perspective. As the left middle temporal gyrus has been specifically related to the factor of perspective, this correlation might speculatively indicate cognitive perspective taking during the observation of psychologically painful interactions in order to evaluate the social situation. Since patients with BPD showed reduced cognitive perspective taking, the mechanism for evaluating social situations might be altered.

A similar theory has been also postulated by Ducasse and colleagues, who proposed that a deficient regulatory control system, which operates through anterior brain regions, would led to failure in top-down inhibition and finally over-interpretation when patients with BPD are experiencing psychological pain [65]. How the above-mentioned concepts could be transferred from somatic and psychological pain processing to processing of empathy for psychological and somatic pain warrants further investigations. When considering the correlational findings, in our previous study, we observed that the impact of childhood maltreatment on empathy for psychological pain (on the behavioural level) has been mediated by alexithymia [24], which brings the role of childhood maltreatment in altered social functioning into account. Here, we replicated the previous findings by showing correlations between empathy for pain ratings for psychological pain and CTQ scores (supplementary information Table S5). In addition, correlations between activation of the inferior triangular part of the frontal gyrus during viewing psychologically painful interactions and childhood maltreatment were observed, which further supports the idea of childhood experiences being relevant for social information processing and behaviour and BPD psychopathology.

Limitations include the female-only sample and the task design for female participants, limiting generalizability; the use of still images restricting ecological validity; and only 24% of patients being medication-free, which may influence the results. Another limitation is the lower education level in the patient group, since it is known that socioeconomic factors might affect compassion [66] and prosocial behaviour [67]. Moreover, the inclusion of several psychology students in the control group may have affected the study’s outcomes due to their prior experience with experimental tasks. Even if effect sizes for the present behavioural findings were large, psychological findings should be generally interpreted and compared with caution, as many studies suffer from small effect sizes and insufficient statistical power, a problem that has been shown to undermine the reliability of findings in neuroscience and psychology [68]. Finally, although the hypotheses were specified a priori, the study was not preregistered, which may reduce transparency. Additionally, the results of the exploratory analysis should be interpreted with caution.

In summary, BPD patients show increased pain ratings for neutral and psychological pain and elevated pain thresholds. These findings constitute a replication of previous research. For the first time, we further showed reduced activations for clusters including the right insula (and hippocampus), bilateral superior and middle frontal (med) gyrus, left middle temporal gyrus, left pre-and postcentral gyri, left Putamen and right anterior cingulum in the patients group during processing of social interactions. Although not specific for BPD, activation of the left middle temporal gyrus during observation of neutral scenarios correlated negatively with alexithymia and self-harm behaviour, suggesting potential regulatory dysfunction. Since brain activation during psychological pain processing in the same region was related to perspective taking in healthy controls, lower cognitive perspective taking could be implicated in altered processing and evaluation of psychological pain in individuals with BPD. Finally, using the SIET in fMRI, we further demonstrated that psychological and somatic pain evoke distinct brain activation patterns compared to neutral social interactions, with psychological pain eliciting highest responses in left cuneus, the right calcarine sulcus and bilateral precuneus, and somatic pain showing strongest activation in left middle temporal gyrus and left inferior parietal lobule.

## Supplementary information

Supplementary Information

## Data Availability

The data from this study are available upon request. Enquiries should be directed to the corresponding author.
