# Supplementary material for: Reduced activation in empathy core regions during observation of social interactions in patients with borderline personality disorder: an fMRI-study
Source: Transl Psychiatry. 2026 Apr 3;16:232. doi: 10.1038/s41398-026-03989-5 (PMC13066390; doi:10.1038/s41398-026-03989-5)
Supplement: Supplementary file 1 — Supplementary Information [file 41398_2026_3989_MOESM1_ESM.docx]

**Supplementary Information**

**Extended methods**

**Questionnaires**

The Edinburgh Handedness Inventory ^31^ was used to calculate a laterality quotient = (sum of right-handed response-sum left handed response)/(sum of all responses) x 100. The Pain Sensitivity Questionnaires assessed how much pain participants would feel in a situation on a numeric rating scale from 0 (no pain at all) to 10 (10 = worst pain imaginable). Seven items were averaged for the scale ‘minor pain’ and seven items were averaged for the ‘moderate pain’ scale. The PSQ- total score was calculated as the average of all ratings of painful situations. For the Interpersonal-Reactivity-Index (IRI), two cognitive subscales, perspective taking (PT) and fantasy (FS), and two affective subscales, empathic concern (EC) and personal distress (PD) were calculated by summing up the respective items participants rated on a 5- point Likert scale from 0 (that does not describe me well) to 4 (that describes me very well) (German version with 31 items ^34^). The Toronto Alexithymia Scale-20 (TAS-20; German Version ^36^) contains 20 questions that participants rated using a 5-point Likert scale (1 = strongly disagree, 5 = strongly agree). Following the model of Bagby et al. ^35,^ a three-factor model with difficulty identifying feelings (DIF), difficulty describing feelings (DDF), and externally oriented thinking (EOT) was used. The Childhood Trauma Questionnaire (CTQ; German Version ^37^) consists of 28 questions belonging to the five subscales emotional abuse, physical abuse, sexual abuse, emotional neglect, and physical neglect. Subjects rated statements on a 5-point Likert scale from 1 (never) to 5 (very often) and responses were summed up. By using the Borderline Symptom List 23 (BSL-23; ^38^), participants were asked to rate symptoms associated with BPD on a 5-point Likert scale from 0 (not all) to 4 (very strong). Mean scores of all 23 items were calculated. For assessing self-harm behaviour, the Deliberate Self-Harm Inventory (DSHI; ^39^; German version ^40^ was used, which is a self-rating questionnaire assessing the presence, frequency, severity, duration and type of intentional self-harming behaviour without conscious suicidal intention. 17 items asked for the experience of different ways of self-harms and participants were required to rate yes/no. By using 5 additional items, the duration, time and frequency of self-injurious behaviour was recorded. A sum score of all 17 items has been computed. For assessment of depressive symptoms, answers on the Beck Depression Inventory II ^41; 42^ to 21 items (from 0 to 3) were summed up.

**Social interaction empathy task**

Please see Figure S1 for a graphical description of the empathy task


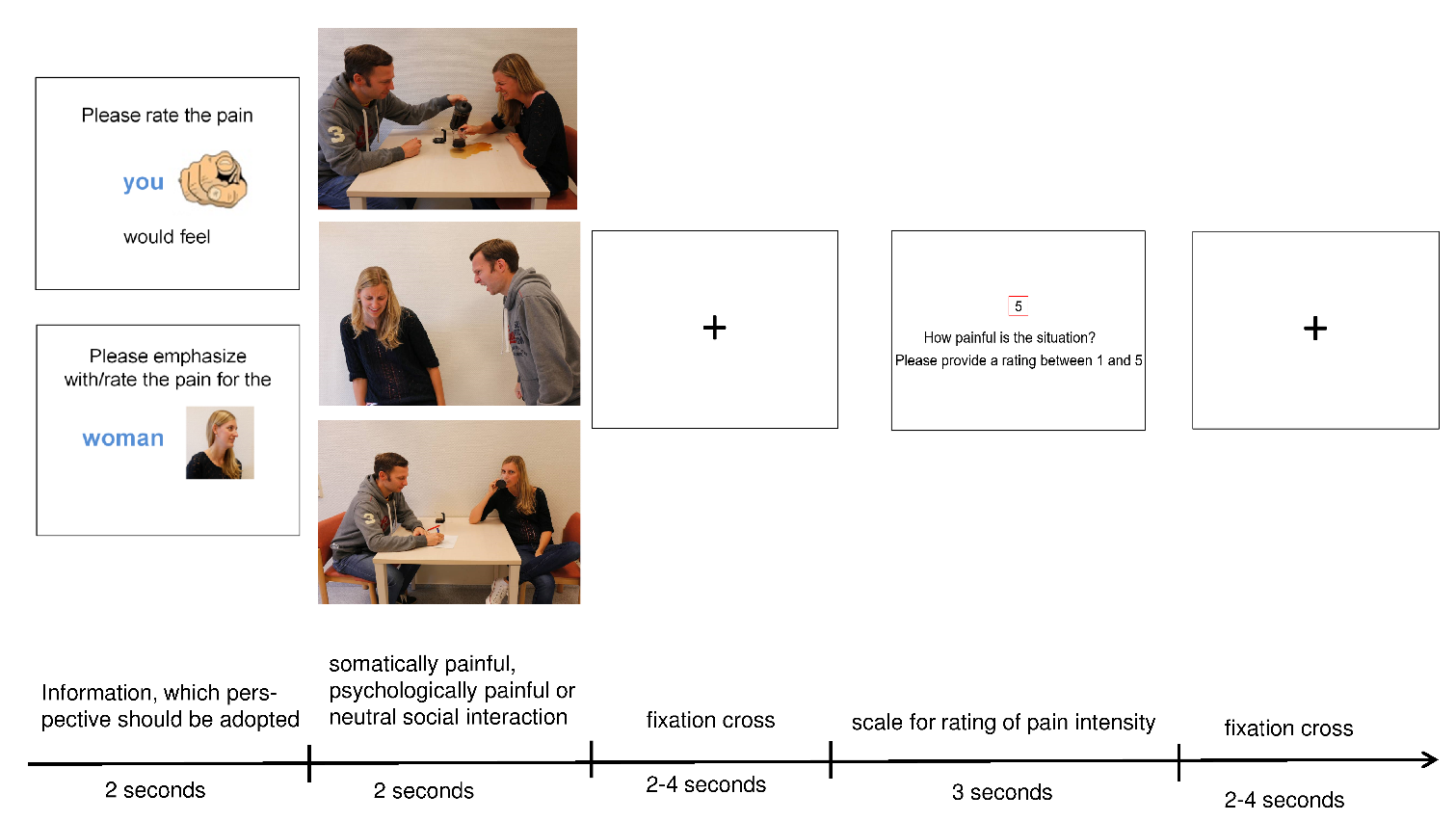


**Figure S1**: Description of the Social Interaction Empathy Task, in an adopted fMRI version.

**Extended results**

**Table S1**: Results of the deliberate-self-harm inventory in control participants and patients with BPD.

| **DSH behaviour** | **HC frequency (no/yes %yes)** | | **BPD frequency (no/yes %yes)** | | **Statistics (Chi-squared test)** |
| --- | --- | --- | --- | --- | --- |
| Cutting | 47/7 | 13.0 % | 4/46 | 92.0 % | *χ*²(1) = 64.90, *p*<0.001 |
| Burning with cigarette | 54/0 | 0.0 % | 37/13 | 26.0 % | *χ*²(1) = 16.05, *p*<0.001 |
| Burning with lighter or match | 52/2 | 3.7 % | 30/19 | 38.8 % | *χ*²(1) = 19.47, *p*<0.001 |
| Carving words | 52/2 | 3.7 % | 28/22 | 44.0 % | *χ*²(1) = 23.75, *p*<0.001 |
| Carving pictures | 54/0 | 0.0 % | 34/16 | 32.0 % | *χ*²(1) = 20.42, *p*<0.001 |
| Severe scratching | 50/4 | 7.4 % | 9/40 | 81.6 % | *χ*²(1) = 57.84, *p*<0.001 |
| Biting | 51/3 | 5.6 % | 32/18 | 36.0 % | *χ*²(1) = 14.93, *p*<0.001 |
| Rubbing sandpaper | 54/0 | 0.0 % | 47/3 | 6.0 % | *χ*²(1) = 3.34, *p*=0.068 |
| Dripping acid | 54/0 | 0.0 % | 49/1 | 2.0 % | *χ*²(1) = 1.09, *p*=0.296 |
| Scraping skin with bleach | 54/0 | 0.0 % | 49/1 | 2.0 % | *χ*²(1) = 1.09, *p*=0.296 |
| Sticking pins and needles | 54/0 | 0.0 % | 18/32 | 64.0 % | *χ*²(1) = 49.92, *p*<0.001 |
| Rubbing glass on skin | 54/0 | 0.0 % | 34/16 | 32.0 % | *χ*²(1) = 17.58, *p*<0.001 |
| Breaking bones | 55/0 | 0.0 % | 49/1 | 2.0 % | *χ*²(1) = 1.11, *p*=0.2921 |
| Banging the head | 55/0 | 0.0 % | 23/27 | 54.0 % | *χ*²(1) = 39.98, *p*<0.001 |
| Punching oneself | 53/2 | 3.6 % | 19/31 | 62.0 % | *χ*²(1) = 41.40, *p*<0.001 |
| Interfering wound healing | 52/3 | 5.3 % | 17/33 | 66.0 % | *χ*²(1) = 42.61, *p*<0.001 |
| Other methods | 54/1 | 1.8 % | 17/33 | 66.0 % | *χ*²(1) = 49.27, *p*<0.001 |
|  |  |  |  |  |  |
| If self-harm has been conducted: | |  |  |  |  |
| When did behaviour start ?(2-3 years/ 4-5 years/ 6 years or longer ago | 2/2/9 |  | 7/3/5/35 |  | *χ*²(3) = 3.24, *p*=0.357 |
| How often did it happen? (1x/ 2x / 3-5x/ >5x) | 3/4/3/3 |  | 2/6/41 |  | *χ*²(3) = 25.82, *p*<0.001 |
| When was the last time? (last year/ 2-3 years/ 4-5 years/ ≥6 years ago | 2/5/1/5 |  | 42/2/1/4 |  | *χ*²(3) = 25.43, *p*<0.001 |
| For how many years did you do that? (up to 1 year/2-3 years/4-5 years/≥6 years) | 9/0/0/3 |  | 5/5/7/31 |  | *χ*²(3) = 22.82, *p*<0.001 |
| Did you had to go to the hospital? (no/yes) | 13/0 |  | 30/19 |  | *χ*²(3) = 7.27, *p*=0.007 |
| Total number of DSHI-items answered with yes (mean (*SD*), median)* | 0.46 (0.99), 27.95 | | 7.04 (2.93),79.01 | | *U*=24.5, *Z*=-8.91, *p*<0.001 |

** Comparison by Mann-Whitney U-test*

**Social Interaction Empathy Task**

The perspective x condition x group interaction mainly showed that patients with BPD exhibited a heightened pain rating of psychological pain when assessing their own experiences in comparison to the third-person perspective. In contrast, healthy controls assigned a higher psychological pain rating in the third-person perspective compared to the first-person perspective (see Table S2).

**Table S2**: Post-hoc comparisons for the interaction of condition x perspective x group.

| **Condition** | **Pers-pective** | **HC (n=53)**  **M (*SD*)** | **BPD (n= 49) M (*SD*)** | **BPD vs. HC** | **1^st^ vs 3^rd^ PP** |
| --- | --- | --- | --- | --- | --- |
| Neutral | 1^st^ PP | 1.16 (0.21) | 1.61 (0.73) | *t*(55.48)=-4.17, *p*<0.001, *d*=-0.85 | HC: *t*(52)=-1.96, *p*=0.055, *d*=-0.27  BPD: *t*(48)=1.12, *p*=0.268, *d*=0.16 |
|  | 3^rd^ PP | 1.20 (0.28) | 1.56 (0.64) | *t*(64.08)=-3.56, *p*<0.001, *d*=-0.73 |  |
| Psycholo-gical pain | 1^st^ PP | 2.98 (1.03) | 4.07 (0.67) | *t*(90.03)=-6.41, *p*<0.001, *d*=-1.25 | HC: *t*(52)=-3.57, *p*<0.001, *d*=-0.49  BPD: *t*(48)=5.10, *p*<0.001, *d*=0.73 |
|  | 3^rd^ PP | 3.14 (0.91) | 3.71 (0.64) | *t*(93.27)=-3.68, *p*<0.001, *d*=-0.72 |  |
| Somatic pain | 1^st^ PP | 3.47 (0.89) | 3.21 (0.70) | *t*(97.53)=1.65, *p*=0.102, *d*=0.32 | HC: *t*(52)=-4.75, *p*<0.001, *d*=-0.65  BPD: *t*(48)=-6.69, *p*<0.001, *d*=-0.96 |
|  | 3^rd^ PP | 3.66 (0.86) | 3.78 (0.55) | *t*(89.67)=-0.83, *p*=0.410, *d*=-0.16 |  |

*1^st^ PP = first-person perspective; 3^rd^ PP = third-person perspective*

The main effect perspective indicates that social situations were rated as more painful in the 3^rd^ PP compared to the 1^st^ PP, independent of condition and group (1^st^ PP: *M*=2.7 (0.6); 3^rd^ PP: *M*=2.8 (0.5); *t*(101)=-4.0, *p*<0.001, *d*=-0.40). The Interaction condition x perspective shows differences in pain ratings between perspectives only for somatically painful interactions (see Table S3 and Figure S2) in the whole sample. In addition, ratings for psychological pain differed from ratings for somatic pain only in the 3^rd^ PP (psychological pain vs somatic pain: 1^st^ PP *t*(101)=1.36, *p*=0.177, *d*=0.14; 3^rd^ PP *t*(101)=-3.47, *p*<0.001, *d*=-0.34).

**Table S3**: Posthoc comparisons for the interaction of condition x perspective.

| **Condition** | **Perspective** | **Pain rating M (*SD*)**  **N = 102** | **1^st^ vs 3^rd^ PP** |
| --- | --- | --- | --- |
| Neutral | 1^st^ PP | 1.38 (0.57) | *t*(101)=-0.22, *p*=0.830, *d*=0.02 |
|  | 3^rd^ PP | 1.37 (0.52) |  |
| Psychological pain | 1^st^ PP | 3.51 (1.03) | *t*(101)=1.92, *p*=0.058, *d*=0.19 |
|  | 3^rd^ PP | 3.41 (0.84) |  |
| Somatic pain | 1^st^ PP | 3.35 (0.81) | *t*(101)=-7.52, *p*<0.001, *d*=-0.74 |
|  | 3^rd^ PP | 3.72 (0.73) |  |

**
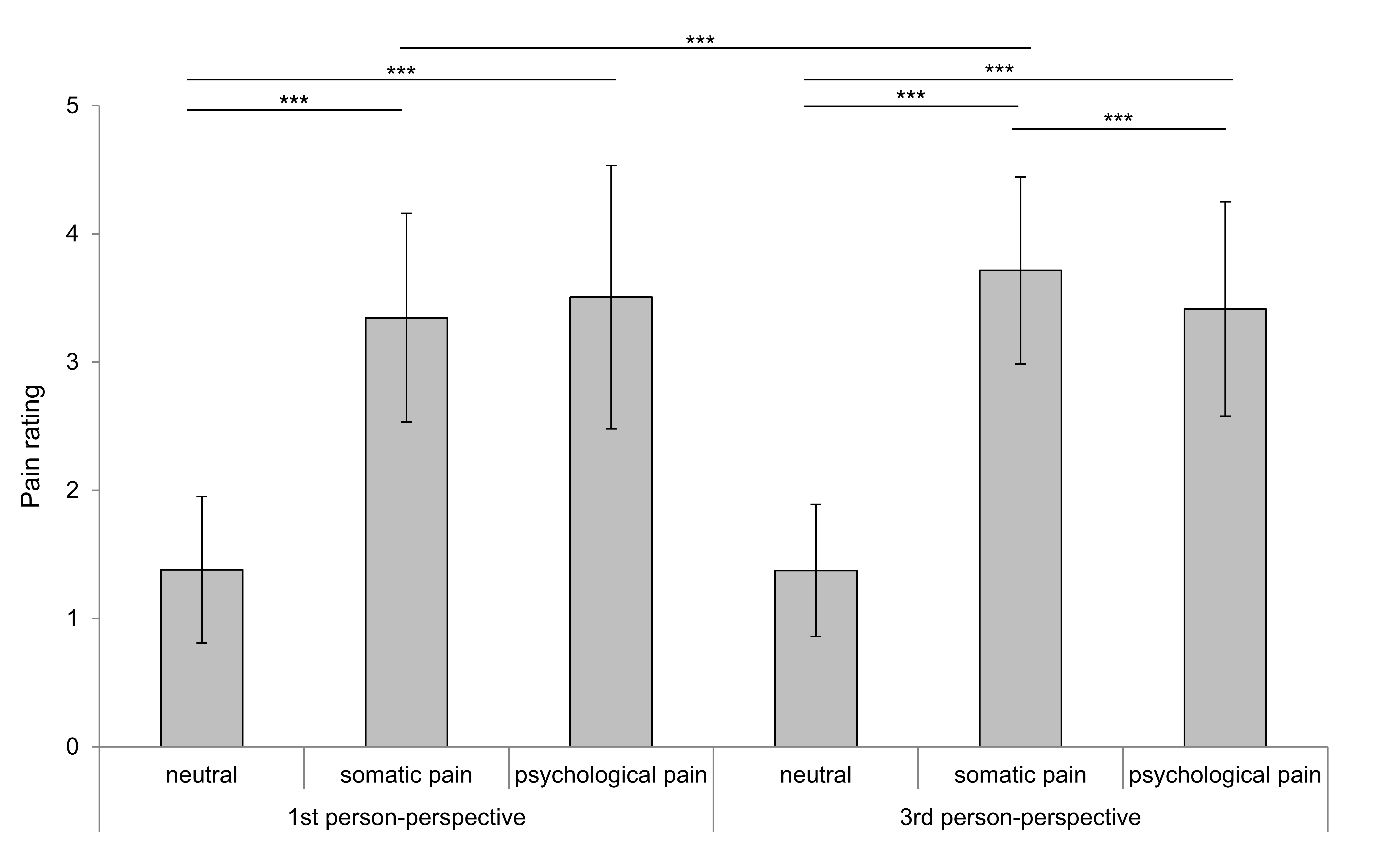
**

**Figure S2**: Graphical presentation of differences between perspectives for neutral, psychologically painful and somatically painful conditions. Error bars indicate *SD,* ****p*<0.001.

**fMRI Data**

**Table S4:** Brain activations during the SIET with an Initial threshold of *p*[FWE] < 0.001 for k > 10 voxel for the main effects of group, condition and perspective.

| **Main effect** | **Region** | **Hemisphere** | **Coordinates (MNI)** | **Extent (voxel) region (total cluster)** | ***F* (peak)** | **equiv*Z* (peak)** |
| --- | --- | --- | --- | --- | --- | --- |
| Group | | | | | | |
|  | Hippocampus | R | 24 -22 -6 | 11 (74) | 62.15 | 7.582 |
|  | Insula | R | 40 -10 -12 | 5 (32) | 43.91 | 6.393 |
|  | Insula | R | 40 2 -8 | 16 (22) | 37.77 | 5.930 |
|  | Superior frontal gyrus | R | 12 24 52 | 55 (85) | 58.60 | 7.369 |
|  | Middle temporal gyrus | L | -54 -58 6 | 53 (53) | 49.24 | 6.767 |
|  | Superior temporal Pole | R | 34 10 -22 | 11 (15) | 47.14 | 6.622 |
|  | Postcentral gyrus | L | -46 -8 32 | 24 (51) | 43.85 | 6.389 |
|  | Superior medial frontal gyrus | L | -10 34 48 | 29 (56) | 41.01 | 6.179 |
|  | Middle frontal gyrus | L | -26 50 22 | 12 (22) | 40.78 | 6.162 |
|  | Middle frontal gyrus | R | 32 44 -2 | (13) | 37.77 | 5.930 |
|  | Hippocampus | L | -34 -8 -20 | 10 (16) | 40.58 | 6.147 |
|  | Insula | L | 32 -20 4 | 6 (19) | 39.61 | 6.073 |
| Condition | | | | | | |
|  | Supramarginal gyrus | L | -60 -28 28 | 243 (332) | 52.30 | 65535.00 |
|  | Middle temporal gyrus | L | -50 -62 -4 | 35 (49) | 25.79 | 6.61 |
|  | Cuneus | L | -18 -60 18 | 26 (49) | 23.94 | 6.35 |
|  | Calcarine sulcus | R | 20 -60 20 | 22 (44) | 23.62 | 6.31 |
|  | inferior triangular part of  the frontal gyrus | L | -48 24 4 | 17 (17) | 21.22 | 5.95 |
|  | inferior parietal lobule | L | -40 -42 50 | 20 (20) | 19.67 | 5.71 |
| Perspective | |  |  |  |  |  |
|  | Middle temporal gyrus | L | -56 -36 0 | 23 (26) | 34.98 | 5.71 |

*Only regions contributing most to the clusters are listed. Cluster sizes for those regions are provided as well as total cluster sizes.*

**Correlations between childhood maltreatment and fMRI data**

Correlations between the levels of adverse experiences during childhood and brain activations as well as pain ratings during the SIET are shown in Table S5.

**Table S5:** Correlations (*r*(*p*)) between brain activations during images showing painful situations and childhood maltreatment assessed by using the Childhood Trauma Questionnaire in the whole sample. Activations extracted were identified by the main effect of condition. Significant correlations are marked by bold font.

| **Region/ Pain** | | **Emotional abuse** | **Physical abuse** | **Sexual abuse** | **Emotional neglect** | **physical neglect** |
| --- | --- | --- | --- | --- | --- | --- |
| **Brain activations during the SIET** | | | |  |  |  |
| Supramarginal L | | | |  |  |  |
|  | somatic pain | 0.095 (0.361) | 0.082 (0.430) | 0.146 (0.159) | 0.097 (0.352) | 0.107 (0.300) |
|  | psychological pain | 0.094 (0.365) | 0.062 (0.551) | 0.114 (0.270) | 0.066 (0.526) | 0.043 (0.678) |
| Temporal mid L | | | |  |  |  |
|  | somatic pain | -0.016 (0.877) | 0.148 (0.151) | 0.126 (0.224) | 0.121 (0.244) | **0.223 (0.030)** |
|  | psychological pain | -0.006 (0.954) | 0.115 (0.268) | 0.101 (0.330) | 0.142 (0.168) | **0.223 (0.030)** |
| Cuneus L | | | |  |  |  |
|  | somatic pain | 0.125 (0.226) | 0.018 (0.864) | -0.028 (0.789) | 0.026 (0.804) | 0.016 (0.878) |
|  | psychological pain | 0.097 (0.349) | -0.051 (0.626) | 0.000 (0.998) | 0.049 (0.634) | 0.003 (0.978) |
| Calcarine R | | | |  |  |  |
|  | somatic pain | -0.009 (0.930) | -0.144 (0.165) | 0.015 (0.883) | -0.039 (0.707) | -0.045 (0.667) |
|  | psychological pain | -0.111 (0.285) | -0.194 (0.060) | -0.139 (0.179) | -0.143 (0.167) | -0.104 (0.316) |
| Frontal inf Tri L | | | | |  |  |
|  | somatic pain | **-0.214 (0.037)** | **-0.212 (0.039)** | -0.022 (0.832) | -0.193 (0.061) | -0.106 (0.306) |
|  | psychological pain | **-0.384 (<0.001)** | **-0.388 (<0.001)** | -0.198 (0.054) | **-0.299 (0.003)** | **-0.334 (<0.001)** |
| Parietal Inf L | | | | |  |  |
|  | somatic pain | 0.019 (0.854) | -0.022 (0.832) | 0.015 (0.886) | 0.055 (0.594) | 0.155 (0.134) |
|  | psychological pain | -0.006 (0.952) | -0.113 (0.276) | -0.040 (0.701) | 0.066 (0.524) | 0.076 (0.465) |
| Precuneus L | |  |  |  |  |  |
|  | somatic pain | 0.010 (0.927) | 0.101 (0.332) | 0.025 (0.811) | -0.109 (0.293) | 0.006 (0.954) |
|  | psychological pain | 0.052 (0.620) | 0.082 (0.430) | 0.069 (0.507) | 0.128 (0.218) | 0.179 (0.082) |
| **Pain ratings during the SIET** | | | | | | |
|  | neutral | **0.349 (<0.001)** | 0.192 (0.054) | 0.257 (0.009) | **0.317 (0.001)** | **0.231 (0.020)** |
|  | somatic pain | -0.013 (0.894) | 0.088 (0.380) | 0.143 (0.150) | -0.027 (0.786) | -0.066 (0.508) |
|  | psychological pain | **0.390 (<0.001)** | **0.231 (0.020)** | **0.283 (0.004)** | **0.388 (0.001)** | **0.271 (0.006)** |

**Correlations between behavioural and fMRI data**

Correlations between pain rating during the SIET and brain activations during the SIET are shown in Table S6 for pain ratings with perspectives averaged and in S7 for perspectives separated. In Table S8, correlations between brain activations and pain thresholds and evaluation during the pain-pressure test are presented.

**Table S6:** Correlations (r(p)) between brain activations during the SIET (regions indicated by main effect of group) and pain ratings (PR) during the SIET in the whole sample, patients with BPD and healthy controls separately (controlled for education). Activations for specific conditions were correlated with the respective pain ratings. Significant correlations are marked by bold font.

|  | **PR neutral** | | | **PR somatic pain** | | | **PR psychological pain** | | |
| --- | --- | --- | --- | --- | --- | --- | --- | --- | --- |
|  | **all** | **BPD** | **HC** | **all** | **BPD** | **HC** | **all** | **BPD** | **HC** |
| Insula R | **-0.239 (0.021)** | -0.085 (0.589) | **-0.340 (0.017)** | 0.101 (0.337) | -0.061 (0.699) | 0.164 (0.261) | -0.199 (0.056) | -0.038 (0.809) | -0.143 (0.327) |
| Superior frontal R | 0.095 (0.364) | **0.340 (0.026)** | 0.078 (0.592) | **0.206 (0.048)** | 0.177 (0.257) | 0.215 (0.137) | -0.133 (0.202) | -0.127 (0.418) | 0.102 (0.485) |
| Temporal mid L | **-0.239 (0.021)** | -0.181 (0.245) | -0.162 (0.265) | 0.067 (0.524) | 0.026 (0.871) | 0.081 (0.579) | -0.076 (0.468) | 0.036 (0.817) | -0.010 (0.947) |
| Precentral L | -0.173 (0.096) | -0.007 (0.964) | -0.227 (0.116) | 0.053 (0.612) | 0.121 (0.441) | -0.032 (0.827) | -0.194 (0.063) | -0.170 (0.277) | -0.047 (0.749) |
| Superior frontal (med) L | 0.164 (0.117) | 0.293 (0.056) | 0.119 (0.417) | 0.166 (0.112) | 0.206 (0.186) | 0.126 (0.388) | -0.080 (0.448) | 0.126 (0.422) | 0.016 (0.913) |
| Frontal mid L | -0.077 (0.465) | 0.051 (0.747) | 0.056 (0.704) | 0.108 (0.303) | 0.129 (0.411) | 0.072 (0.621) | -0.118 (0.260) | 0.228 (0.142) | -0.185 (0.202) |
| Putamen L | -0.117 (0.265) | 0.148 (0.345) | **-0.446 (0.001)** | 0.089 (0.395) | 0.020 (0.899) | 0.124 (0.397) | **-0.296 (0.004)** | -0.080 (0.609) | **-0.390 (0.006)** |
| Frontal mid R | 0.114 (0.276) | **0.311 (0.042)** | 0.149 (0.307) | 0.047 (0.652) | 0.116 (0.459) | -0.034 (0.815) | -0.073 (0.489) | 0.082 (0.600) | 0.009 (0.948) |
| Ant Cingulum R | -0.017 (0.869) | 0.193 (0.216) | -0.187 (0.198) | 0.150 (0.151) | 0.106 (0.498) | 0.207 (0.153) | -0.108 (0.301) | -0.160 (0.306) | 0.070 (0.633) |

**Table S7:** Correlations (*r*(*p*)) between brain activations during images showing painful situations and pain ratings during the SIET in the whole sample (controlled for education levels). Activations for specific conditions and perspectives were correlated with the respective pain ratings and were identified by the main effect of group. Significant correlations are marked by bold font.

|  | **1^st^ person perspective: Pain rating** | | | **3^rd^ person perspective: Pain rating** | | |
| --- | --- | --- | --- | --- | --- | --- |
| Region | **neutral** | **somatic pain** | **psychological pain** | **neutral** | **somatic pain** | **psychological pain** |
| Insula R | -0.053 (0.616) | 0.100 (0.339) | -0.128  (0.223) | **-0.260 (0.012)** | 0.058 (0.578) | **-0.214**  **(0.039)** |
| Superior frontal R | -0.004 (0.973) | 0.187 (0.072) | **-0.216**  **(0.038)** | 0.157 (0.134) | 0.109 (0.298) | 0.035  (0.741) |
| Temporal mid L | **-0.250 (0.016)** | 0.055 (0.599) | -0.120  (0.252) | -0.203 (0.051) | 0.046 (0.664) | -0.004  (0.968) |
| Precentral L | -0.081 (0.439) | 0.043 (0.680) | **-0.229**  **(0.027)** | **-0.231 (0.026)** | 0.005 (0.966) | -0.118  (0.259) |
| Superior frontal (med) L | 0.079 (0.454) | 0.167 (0.109) | -0.109  (0.298) | 0.172 (0.099) | 0.031 (0.765) | 0.008  (0.943) |
| Frontal mid L | -0.139 (0.185) | 0.098 (0.350) | -0.068  (0.518) | -0.012 (0.908) | 0.026 (0.804) | -0.109  (0.296) |
| Putamen L | -0.105 (0.315) | 0.035 (0.739) | **-0.268**  **(0.009)** | -0.103 (0.328) | 0.073 (0.486) | **-0.223**  **(0.032)** |
| Frontal mid R | 0.064 (0.542) | 0.082 (0.435) | -0.097  (0.354) | 0.118 (0.258) | 0.009 (0.932) | 0.024  (0.819) |
| Ant Cingulum R | -0.012 (0.910) | 0.201 (0.054) | -0.072  (0.492) | -0.027 (0.799) | 0.006 (0.956) | -0.092  (0.381) |

**Table S8:** Pearson correlations (*r*(*p*)) between brain activations during images showing painful situations during the SIET (perspectives averaged) and pain thresholds and evaluation during the PPT (left and right hand results averaged) in the whole sample and HC and BPD groups separately. Activations extracted were identified by the main effect of group. Significant correlations are marked by bold font.

| **Region/ Pain** | | **Pain threshold** | | | **Pain rating** | | | **Unpleasantness rating** | | |
| --- | --- | --- | --- | --- | --- | --- | --- | --- | --- | --- |
|  | | **All** | **BPD** | **HC** | **All** | **BPD** | **HC** | **All** | **BPD** | **HC** |
| Insula R | | | |  |  |  |  |  |  |  |
|  | somatic pain | **-0.229 (0.028)** | -0.257 (0.092) | -0.058 (0.696) | 0.196 (0.061) | 0.138 (0.371) | 0.155 (0.293) | 0.103 (0.330) | 0.134 (0.387) | 0.019 (0.900) |
|  | psychological pain | -0.173 (0.099) | -0.188 (0.222) | -0.008 (0.955) | 0.143 (0.175) | 0.102 (0.511) | 0.091 (0.538) | 0.073 (0.487) | 0.087 (0.576) | 0.000 (1.000) |
| Superior frontal R | | | |  |  |  |  |  |  |  |
|  | somatic pain | -0.035 (0.742) | -0.124 (0.421) | 0.174 (0.236) | **0.288 (0.005)** | 0.211 (0.169) | **0.314 (0.030)** | 0.156 (0.137) | 0.107 (0.491) | 0.173 (0.240) |
|  | psychological pain | 0.059 (0.574) | -0.032 (0.839) | **0.358 (0.012)** | **0.306 (0.003)** | 0.155 (0.314) | **0.370 (0.010)** | 0.177 (0.092) | 0.122 (0.429) | 0.181 (0.220) |
| Temporal mid L | | | |  |  |  |  |  |  |  |
|  | somatic pain | -0.056 (0.599) | 0.040 (0.798) | -0.004 (0.980) | 0.031 (0.766) | 0.173 (0.261) | -0.139 (0.346) | 0.028 (0.793) | 0.122 (0.431) | -0.079 (0.594) |
|  | psychological pain | 0.054 (0.610) | 0.139 (0.367) | 0.113 (0.443) | 0.195 (0.063) | **0.314 (0.038)** | 0.050 (0.735) | 0.159 (0.129) | 0.224 (0.144) | 0.082 (0.578) |
| Precentral L | | | |  |  |  |  |  |  |  |
|  | somatic pain | -0.061 (0.564) | 0.071 (0.645) | -0.058 (0.697) | 0.149 (0.157) | 0.272 (0.074) | -0.061 (0.678) | 0.031 (0.770) | 0.181 (0.241) | -0.190 (0.196) |
|  | psychological pain | -0.022 (0.835) | 0.016 (0.916) | 0.108 (0.464) | 0.124 (0.237) | 0.284 (0.061) | -0.107 (0.470) | 0.057 (0.591) | 0.219 (0.154) | -0.149 (0.311) |
| Superior frontal (med) L | | | | |  |  |  |  |  |  |
|  | somatic pain | -0.042 (0.691) | 0.006 (0.971) | 0.001 (0.993) | **0.252 (0.015)** | **0.381 (0.011)** | 0.052 (0.727) | 0.190 (0.070) | 0.297 (0.050) | 0.025 (0.865) |
|  | psychological pain | 0.019 (0.854) | -0.004 (0.981) | 0.254 (0.082) | **0.263 (0.011)** | **0.303 (0.046)** | 0.140 (0.341) | 0.185 (0.077) | 0.209 (0.174) | 0.112 (0.448) |
| Frontal mid L | | | | | | | | | | |
|  | somatic pain | -0.130 (0.217) | -0.063 (0.682) | -0.101 (0.494) | 0.100 (0.342) | 0.275 (0.071) | -0.133 (0.368) | 0.013 (0.900) | 0.146 (0.345) | -0.163 (0.268) |
|  | psychological pain | -0.076 (0.471) | -0.155 (0.315) | 0.133 (0.368) | 0.062 (0.559) | 0.070 (0.651) | -0.029 (0.845) | 0.002 (0.988) | -0.066 (0.670) | 0.020 (0.893) |
| Putamen L | | | | | | | | | | |
|  | somatic pain | **-0.218 (0.037)** | -0.084 (0.589) | -0.262 (0.073) | 0.007 (0.950) | 0.037 (0.812) | -0.121 (0.413) | -0.107 (0.310) | -0.155 (0.315) | -0.117 (0.429) |
|  | psychological pain | -0.101 (0.338) | -0.059 (0.704) | -0.046 (0.754) | 0.156 (0.138) | 0.186 (0.228) | 0.063 (0.671) | 0.089 (0.401) | 0.007 (0.964) | 0.153 (0.300) |
| Frontal mid R | | | | | | | | | | |
|  | somatic pain | -0.010 (0.926) | 0.006 (0.969) | 0.115 (0.436) | 0.066 (0.531) | 0.294 (0.053) | -0.250 (0.086) | -0.026 (0.807) | 0.132 (0.392) | -0.251 (0.085) |
|  | psychological pain | 0.045 (0.667) | 0.073 (0.636) | 0.191 (0.194) | 0.082 (0.434) | 0.168 (0.276) | -0.106 (0.475) | -0.035 (0.739) | 0.006 (0.971) | -0.149 (0.312) |
| Ant Cingulum R | | | | |  |  |  |  |  |  |
|  | somatic pain | -0.125 (0.235) | -0.124 (0.422) | -0.037 (0.803) | **0.218 (0.037)** | **0.314 (0.038)** | 0.051 (0.733) | 0.104 (0.322) | 0.194 (0.207) | -0.052 (0.725) |
|  | psychological pain | -0.024 (0.823) | -0.110 (0.477) | 0.190 (0.197) | **0.241 (0.020)** | **0.310 (0.040)** | 0.115 (0.438) | 0.192 (0.067) | 0.252 (0.099) | 0.092 (0.533) |
